# Overexpression of Karrikins Receptor Gene *Sapium sebiferum KAI2* Promotes the Cold Stress Tolerance via Regulating the Redox Homeostasis in *Arabidopsis thaliana*

**DOI:** 10.3389/fpls.2021.657960

**Published:** 2021-07-15

**Authors:** Faheem Afzal Shah, Jun Ni, Yuanyuan Yao, Hao Hu, Ruyue Wei, Lifang Wu

**Affiliations:** ^1^Key Laboratory of the High Magnetic Field and Ion Beam Physical Biology, Hefei Institutes of Physical Science, Chinese Academy of Sciences, Hefei, China; ^2^Taihe Experimental Station, Hefei Institutes of Physical Science, Chinese Academy of Sciences, Taihe, China

**Keywords:** karrikins, *KAI2*, cold stress, redox homeostasis, abscisic acid

## Abstract

*KARRIKINS INSENSITIVE2* (*KAI2*) is the receptor gene for karrikins, recently found to be involved in seed germination, hypocotyl development, and the alleviation of salinity and osmotic stresses. Nevertheless, whether *KAI2* could regulate cold tolerance remains elusive. In the present study, we identified that *Arabidopsis* mutants of *KAI2* had a high mortality rate, while overexpression of, a bioenergy plant, *Sapium sebiferum KAI2* (*SsKAI2*) significantly recovered the plants after cold stress. The results showed that the *SsKAI2* overexpression lines (OEs) had significantly increased levels of proline, total soluble sugars, and total soluble protein. Meanwhile, *SsKAI2* OEs had a much higher expression of cold-stress-acclimation-relate genes, such as *Cold Shock Proteins* and *C-REPEAT BINDING FACTORS* under cold stress. Moreover, the results showed that *SsKAI2* OEs were hypersensitive to abscisic acid (ABA), and ABA signaling genes were w significantly affected in *SsKAI2* OEs under cold stress, suggesting a potential interaction between *SsKAI2* and ABA downstream signaling. In *SsKAI2* OEs, the electrolyte leakage, hydrogen peroxide, and malondialdehyde contents were reduced under cold stress in *Arabidopsis*. *SsKAI2* OEs enhanced the anti-oxidants like ascorbate peroxidase, catalase, peroxidase, superoxide dismutase, and total glutathione level under cold stress. Conclusively, these results provide novel insights into the understanding of karrikins role in the regulation of cold stress adaptation.

## Introduction

Plants are the sessile organisms which often exposed to a broad range of adverse environmental conditions. Among a large number of adverse conditions, cold (chilling and freezing) stress significantly limit crop growth and agricultural productivity. Under cold conditions, plants activate their cold resistance mechanism called cold acclimation (Thomashow, 1999; Stockinger et al., 2001; Shi et al., 2014b). Cold acclimation enhances the endogenous as well as inducible components accumulation. The endogenous components, which promote cold tolerance, have extensively been studied and mainly refer to metabolites with anti-oxidant activity (Winkel-Shirley, 2002), with hormonal responses (Eremina et al., 2016) or osmoprotective functions to limit ice nucleation and to overcome the freeze-induced dehydration inside the plant cells (Janská et al., 2010). Furthermore, other regulatory molecules such as polyamines, reactive oxygen species, nitric oxide have also been described to be involved in cold tolerance (Cuevas et al., 2008; Zhao et al., 2009; Puyaubert and Baudouin, 2014; van Buer et al., 2016).

Karrikins, a group of chemical compounds, are present in burnt or charred plant material and its smoke. Karrikins are also produced by the pyrolysis of cellulose and simple sugars (Flematti et al., 2011). To date, natural origin within the plant has not discovered. Karrikins are potent promoters of seed germination of various plants (Flematti et al., 2004). Karrikins promote photomorphogenesis in seedling and negatively regulate the hypocotyl elongation (Nelson et al., 2010). Karrikins inhibited the hypocotyl length under red light, and the length of *Arabidopsis thaliana* seedlings hypocotyl treated with one micromolar KAR_2_ was almost half of the hypocotyl of untreated *Arabidopsis thaliana* seedlings (Nelson et al., 2010; Waters and Smith, 2013). It has also been reported that karrikins might regulate cotyledon expansion and chlorophyll accumulation in the seedlings of *Brassica tournefourtii* and *Lactuca sativa* (Nelson et al., 2010).

Recently, karrikins role against abiotic stresses has also been discovered. For example, it has been found that karrikins might play an essential role in the chilling response of tea plants (Zhao et al., 2012). In tomato, seed primed in butenolide (a karrikin) produced significantly more vigorous seedlings than the water-primed seeds. Vigor indices of seedlings produced by butenolide-primed seeds were significantly higher under different abiotic stresses conditions (salinity, temperature, or osmoticum) compared to control or water-primed seeds (Neeru and Van, 2007). In a bioenergy plant, *Sapium sebiferum*, KAR_1_ has been reported to alleviate osmotic and salinity stresses by regulating redox homeostasis (Shah et al., 2020). *KARRIKINS INSENSITIVE2 (KAI2)*, which encodes an α/β-fold hydrolase, is a receptor gene for karrikins (Scaffidi et al., 2012; Li et al., 2013). Hydrophobic pocket in KAI2 has a conserved catalytic triad (Ser–His–Asp) (Kagiyama et al., 2013) and, KAI2 also has a hydrolyzes pocket (Hamiaux et al., 2012), which may bind to the karrikins (Boyer et al., 2012; Hamiaux et al., 2012). *KAI2* was reported to be involved in the stomatal closure, regulation of cuticle formation, membrane integrity, and anthocyanin biosynthesis, which contributes to plant alleviation to the osmotic stress (Li et al., 2017). Recently, it has been reported that the karrikins-*KAI2* signaling system provided stress tolerance by inhibiting germination in *Arabidopsis* under unfavorable conditions (Wang et al., 2018).

In this study, the homologous gene of *SsKAI2* was identified in an ornamental and bio-energetic woody perennial plant *Sapium sebiferum* and characterized in *Arabidopsis thaliana* under cold stress. After finding the *SsKAI2* alleviation of the cold stress tolerance in *Arabidopsis*, we conducted several experiments to find out the possible mechanism in the *SsKAI2* improved *Arabidopsis* under cold stress.

## Materials and Methods

### SsKAI2 Gene Cloning, Bioinformatics Analysis, Vector Construction, and Obtaining Atkai2 Mutants

Full sequance of *SsKAI2* was found by local blasting amino acids sequance of *Arabidopsis* KAI2 in Blast-2.2.31. *S. sebiferum* flower-bud transcriptome (Yang et al., 2015) (Accession: SRX656554)^1^ was used to built a local blast library. Bio-informatics analysis of *SsKAI2* is given in Supplementary Figure 1. The full cDNA sequence of all genes with the translated amino acid sequence is given in Supplementary Data Sheet 1. The full-length open reading frame (ORF) of the *SsKAI2* gene was found by the NCBI ORF finding tool. Neighbor-Joining method was used to built the evolutionary history (Saitou and Nei, 1987). The bootstrap consensus tree built from 500 replicates (Felsenstein, 1985) is representing the taxa evolutionary history. Branches matching to partitions reproduced in <50% bootstrap replicates were distorted. The evolutionary distances were calculated by using the p-distance method (Nei and Kumar, 2000) and were represented in the number of amino acid differences per site. The analysis involved 30 amino acid sequences. All positions containing missing data or gaps were excluded. In the final dataset, there were 249 positions. Evolutionary analyses were conducted in MEGA7 (Kumar et al., 2016).

Gene-specific primers were designed by Primer Premier 5 to amplify the full-length ORF of *SsKAI2* (Supplementary Table 1). The ORF of the *SsKAI2* gene was sequenced from Sangon Biotech (Shanghai) Co., Ltd. Cloned gene sequence double digested at *Sal1* from start and *Sma1* from stop codon site. Full-length ORF of *SsKAI2* was inserted into the expression vector pOCA30 under the control of the CaMV35S promoter, and the resulting *35S:SsKAI2* plasmid was transformed into the Agrobacteria EHA105 strain. The floral dip method was performed for the transformation of the recombinant expression vector in *Arabidopsis*. *Atkai2* mutants, previously described in Waters et al. (2012), were gifted by Dr. Jiayang Li from the Chinese Academy of Sciences.

### Plant Materials and Growth Conditions

*Sapium sebiferum* seedlings were established by our previously developed method (Shah et al., 2018). The seed of the *Arabidopsis* Columbia-0 (Col-0) genotype was obtained from the *Arabidopsis* Biological Resources Center (Columbus, OH, United States). *SsKAI2* was identified, cloned, transformed to *Arabidopsis*, and homozygous *SsKAI2* OEs lines were selected for further experiments. Seeds of wild-type and *SsKAI2* OEs were surfaces sterilized with 70% (v/v) ethanol for 2 min, then incubated in 10% (v/v) sodium hypochlorite (NaClO) for 10 min at room temperature, and washed thrice with double distilled water. The sterilized seeds were plated on ½ Murashige and Skoog (MS) medium supplemented with 1% (w/v) sucrose and 0.8% (w/v) agar and placed at 4 degrees Celsius (°C) for 2 days. Seeds were germinated in a growth room 16/8 h (day/night) photoperiod at 22°C. Seven-day-old *Arabidopsis* seedlings were transferred from ½ MS medium to the soil and grown in a chamber at 22°C, with 16/8 h (long-day conditions) photoperiods, approximately 120 μmol/m^2^/s radiation strength, and 75% humidity.

### Cold Treatment

Cold resistant plants have developed a coping mechanism called cold acclimation (Thomashow, 1999). Cold acclimation mechanism includes the accumulation of soluble sugars (Guy and Huber, 1992), and proline (Verbruggen and Hermans, 2008), stimulation of antioxidants activity, and changes in the plant transcriptome and proteome (Thomashow, 1999; Zuther et al., 2019). Cold acclimation makes the plants ready in low temperatures to face the upcoming freezing temperatures. So, for phenotypical analysis under cold stress, 5-day-old *Arabidopsis* seedlings were cold acclimatized to 4°C for 12 h and then subjected to cold treatment at −20°C for an hour. The plants were again kept at 4°C for 12 h, the plants were then shifted to a plant growth room with a 16/8 h photoperiod at 22°C, approximately 120 μmol photons/m^2^/s, and 75% humidity. The recovery rate was measured 10 days after the cold-shock treatment. Photographs were taken by a Nikon D90 having Nikon DX AF-S NIKKOR 18-105 mm lens (Nikon Corporation, Tokyo, Japan).

### Electrolyte Leakage Measurement

Electrolyte leakage was determined by the previously reported method in the study of Nishiyama et al. (2011). In detail, after placing 15-day-old plants at 0–, – 4−, – 8−, – 12−, – 16−, and −20°C for an hour, five leaves of different plants of each genotype were collected, then plant samples were shifted to the 50 mL tubes containing 40 mL of double distilled water for 24 h. The electric conductivity (EC) of water was determined by the electric conductivity meter. The tubes having 40 ml of water were autoclaved for 20 min at 121°C, and the EC was measured again. The following equation calculated the percentage of electrolyte leakage.

$$\begin{array}{c} \text{Electrolyte}\\ \mathrm{leakage}\left(\%\right) \end{array}=\frac{\mathrm{Electric}\mathrm{conductivity}\mathrm{before}\mathrm{autoclave}}{\mathrm{Electric}\mathrm{conductivity}\mathrm{after}\mathrm{autoclave}}\times 100$$

### Biochemical Analysis

For biochemical analysis, each line of every genotype was subjected to cold acclimation temperature (4°C). The samples were randomly taken from the leaves of five plants of each treatment after 0 (control at 22°C), 3−, 6−, and 12 h of cold treatment (4°C). Samples were immediately frozen in liquid nitrogen, the stored in to −80°C. The total proline, total soluble sugar (TSS), total soluble protein (TSP), hydrogen peroxide (H_2_O_2_), malondialdehyde (MDA), total glutathione (GSH), peroxidase (POD), superoxide dismutase (SOD), ascorbate peroxidase (APX), and catalase (CAT) contents were determined by using a Proline assay kit, a plant soluble sugar content test kit, a total protein quantitative assay kit, an H_2_O_2_ assay kit, an MDA assay kit, a T-GSH assay kit, a POD assay kit, a SOD assay kit, an APX assay kit, and a CAT assay kit (Nanjing Jiancheng Bioengineering Institute, Nanjing, China), respectively, as previously described by Ni et al. (2018).

### Stomata Analyses

Epidermal peels from mature leaves removed with forceps and were incubated in MES/KCl (2-(N-morpholino) ethanesulfonic acid/potassium chloride) buffer supplemented with 0, 10, 30, and 50 μM ABA for 2 h. Stomata were visualized under an epifluorescent microscope using 100× lenses (Eisele et al., 2016). Stomatal aperture was measured by analyzing pictures in ImageJ 1.52a.

### RNA Extraction and Quantitative Real-Time PCR (qPCR)

Fifteen-day-old *Sapium sebiferum* seedlings were subjected to cold stress (4°C), salt stress (200mM NaCl), and osmotic stress (300mM mannitol). The samples were randomly taken from the leaves of five plants of each treatment after 0 h (control at 22°C), 3−, 6−, and 12 h of. Samples were immediately frozen in liquid nitrogen, and stored in to −80°C. *S. sebiferum* flower-bud transcriptome (Accession: SRX656554, see text footnote 2) (Yang et al., 2015) was used to build the local blast library in blast-2.2.31. The full sequences of all genes were searched by local blasting *Arabidopsis* amino-acids sequence. Full-length mRNA sequences of *SsKAI2* is available in Supplementary Data Sheet 1. Primers for quantitative real-time PCR were designed in primer premier 6, and a list of all primers is available in Supplementary Table 1. Expression of cold-acclimation-related genes, ABA-responsive genes under cold stress were investigated in each line after 0 h (control at 22°C), 3−, 6−, and 12 h of cold treatment (4°C). The samples were randomly taken from the leaves of five plants of each treatment. Samples were immediately frozen in liquid nitrogen, the stored in to −80°C. RNA from already frozen and stored samples at −80°C was extracted by E.Z.N.A^®^ plant RNA extraction kit (Omega Bio-tek, Inc., Norcross, GA, United States) using the standard protocol. 500 nanograms of RNA of each sample was reverse transcribed by cDNA Synthesis SuperMix (TransGen Biotech., Shanghai, China) according to the standard protocol. Each cDNA sample was diluted 25 times with double distilled water. The reaction for RT–qPCR was prepared according to the standard protocol of QuantiNova SYBR Green PCR Master Mix (QIAGEN, Pudong, Shanghai, China) then run in the Light Cycler^®^96 (Roche Diagnostics, Indiana, United States). Following the program was set in qPCR: preheating, 95°C for 10 min; amplification (45 cycles) at 95°C for 10 s, at 60°C for 20 s, and 72°C for 20 s; melting curve at 95°C for 2 min, and at 60°C for 30 s, then continuously increased to 95°C. The 2^–ΔΔCt^ method was used to calculate the relative gene expression, as described by Livak and Schmittgen (2001).

### Statistical Analysis

The statistical analyses were done in R Studio 1.1.442. All data were presented in the form of mean ± standard deviation. One-way analysis of variance (ANOVA) was used to test the significant difference between the treatments. The significant difference between the means of different treatments was determined by using the Tukey test at *P <* 0.05.

## Results

### Abiotic Stresses Significantly Induced KAI2 Expression in the *Sapium sebiferum* Seedlings

**FIGURE 1:**
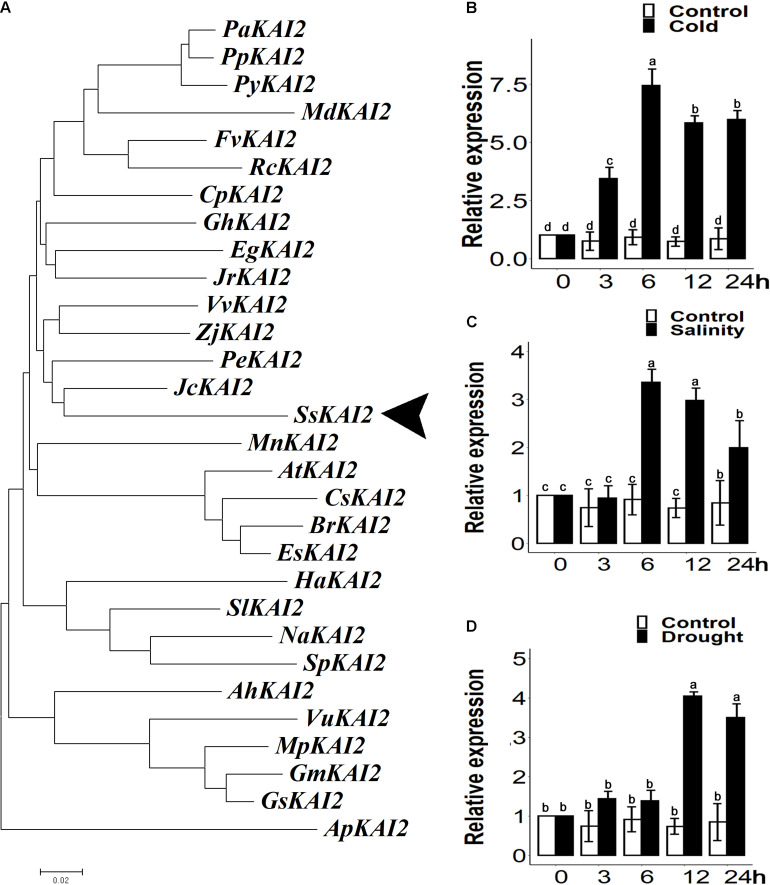
Phylogenetic analysis and relative expression of *KAI2* in *Sapium sebiferum* under cold, salinity, and osmotic stresses. **(A)** Phylogenic analysis of *SsKAI2* protein with its homologs from other species. **(B)** *SsKAI2* relative expression under cold (4°C), salinity (200 mM NaCl) **(C)**, and osmotic stress (300 mM mannitol) **(D)**. Twenty-five-day-old seedlings were used to determine the *SsKAI2* expression level under abiotic stresses. Leaf samples were collected after 3−, 6−, 12−, and 24 h of each treatment. *Sapium sebiferum UBQ10* was used as a reference gene; control treatment at 0 h was considered as 1. qPCR was used to determine gene expression, one-way ANOVA was used to analyze all data, and HSD Tukey’s test was used to perform multiple comparisons at *P* < 0.05 significant level (*n = 3*). Bars with uncommon letters show significant difference at *P* < 0.05.

The *SsKAI2* homolog with 77.8% sequence similarity with *Atkai2* was identified from the *S. sebiferum* transcriptome database (Supplementary Figure 1A). Then, the phylogenetic analysis of the KAI2 protein sequences was carried out from more than 30 plant species (Supplementary Data Sheet 1). The results showed that *SsKAI2* had the highest sequence identity with perennial woody plants, such as *Jatropha caucus* and *Populus euphratica*, which also belong to the Euphorbiaceae family (Figure 1A). We investigated the time-course expression pattern of *SsKAI2* in response to the abiotic stresses (osmotic, salt, and cold) in the 25-day-old *S. sebiferum* seedlings. The results showed that the expression of *KAI2* in *Sapium sebiferum* was increased under cold stress (4°C), salinity (200 mM NaCl), and osmotic stress (300mM mannitol) as compared to control condition (Figures 1B–D). These results suggested that *KAI2* is a stress-responsive gene, which might have a role in the acclimation of abiotic stresses.

### SsKAI2 Overexpression Lines (OEs) Had a Much Higher Survival Rate and Lower Electrolyte Leakage Under Cold Stress

**FIGURE 2:**
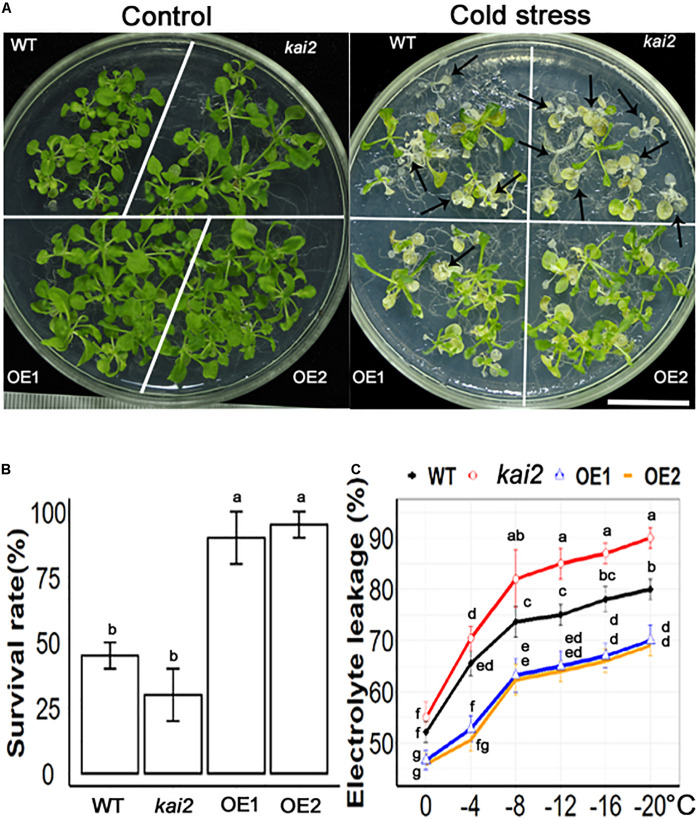
SsKAI2 alleviated cold stress in Arabidopsis. **(A)** Phenotypes of wild-type (WT), *Atkai2*, and *SsKAI2* (OE1 and OE2) *Arabidopsis* at room temperature and under cold stress (−20°C). Pictures were taken after ten days after cold stress, white bar = 2cm. **(B)** Statistical presentation of survival rate after cold stress. **(C)** Electrolyte leakage was measured by subjecting 15-day-old plants at 0−, – 4−, – 8−, – 12−, – 16−, and −20°C for an hour. One-way ANOVA was used to analyzed all data, and HSD Tukey’s test was used to perform multiple comparisons at *P < 0.05* significant level (*n* = 5 in survival rate measurement and *n* = 3 in electrolyte leakage test). Bars or points with uncommon letters showing significant difference at *P < 0.05.*

Cold stress is one of the unfavorable environmental factors that restrict plant growth and development and might cause mortality in the plant. Cold stress alters the structure of the cell membrane, which makes it leaky and results in the loss of ions that are essential for proper functioning in the cell (Uemura et al., 1995). The membrane injury in the plant exposed to cold temperatures is measured by the rises in electrical conductivity resulting from the leakage of the electrolyte from the plant tissues. Exogenous application of KAR_1_ showed 96 ± 3.3% survival rate in *Arabidopsis* under cold stress (Supplementary Figure 2). The results showed that *SsKAI2* OEs, *kai2* mutant, and wild-type *Arabidopsis* started wilting 2 h later cold-shock treatment. On the third day of post-cold-shock treatment, the plants started to regenerate new apical leaves. The recovery rate was recorded on the 10th day after treatment (Figure 2A). The results showed that two overexpression lines *SsKAI2 OE1 and OE2* showed 90 ± 3% and 95 ± 2% recovery rate, respectively, while wild-type was 45 ± 10%, and *kai2* was 30 ± 10% recovered after cold stress (Figure 2B). Further, we determined the electrolyte leakage of *SsKAI2* OEs, *kai2*, and wild-type *Arabidopsis* after subjecting plants in 0−, – 4−, – 8−, – 12−, – 16−, and −20°C for an hour. The results showed that cold stress increased electrolyte leakage in *SsKAI2* OEs, wild-type, and *kai2* plants. Overall, the electrolyte leakage of *SsKAI2* OEs was significantly lower than wild-type plants and *kai2* plants (Figure 2C). These results are suggesting that *KAI2* is involved in the regulation of cold stress alleviation.

### SsKAI2 OEs Had Increased Proline, Total Soluble Sugars, and Proteins Contents Under Cold Stress

**FIGURE 3:**
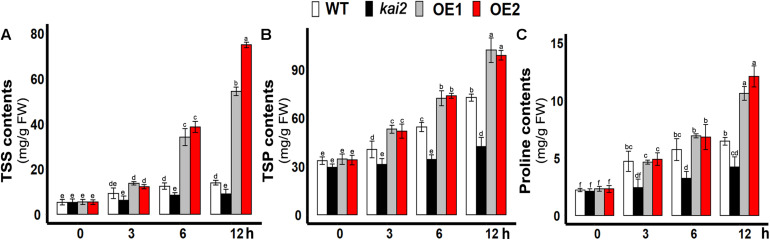
Overexpression of *SsKAI2* promoted TSS and TSP under cold stress. **(A)** Total soluble sugars. **(B)** Total soluble protein contents. **(C)** Proline contents. Twenty-five-day-old plants were subjected to cold treatment. The samples were taken randomly from the leaves of three plants of each independent [(wild-type (WT), *Atkai2*, *KAI2* over-expressed line 1 (OE1) and line 2 (OE2)] line after 3−, 6−, and 12 h under cold treatment (4°C). One-way ANOVA was used to analyzed all data, and HSD Tukey’s test was used to perform multiple comparisons at *P < 0.05* significant level (*n* = 3). An “h” at the *X*-axis of each graph represents time in hours under cold stress. Bars with uncommon letters showing significant difference at *P < 0.05.*

Under abiotic stresses, the accumulation of the total soluble sugars is one of the primary acclimation symptoms. Then sugars modulate the expression of both abiotic and biotic stress-related genes in plants (Barau et al., 2015; Tarkowski and van den Ende, 2015). In this study, total soluble proteins (TSP), the total soluble sugars (TSS), and proline content in the leaves of different *Arabidopsis* lines were determined under cold stress. The results showed that the levels of TSS, TSP, and proline were all significantly increased in both *SsKAI2* OEs in comparison with wild-type and *kai2* mutant (Figures 3A,B). The results suggested that *KAI2*-regulated immediate induction of endogenous metabolites might play an important role in conferring the cold stress acclimation in *Arabidopsis*.

### SsKAI2 OEs Had Lower Hydrogen Peroxide (H_2_O_2_) and Malondialdehyde (MDA) Level Under Cold Stress

**FIGURE 4:**
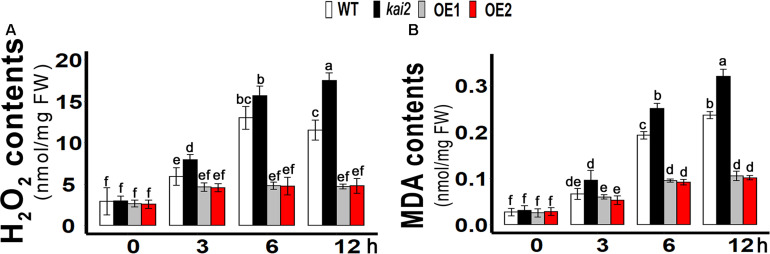
Overexpression of *KAI2* in *Arabidopsis* reduced H_2_O_2_ and MDA levels under cold stress. **(A)** H_2_O_2_ contents. **(B)** MDA contents. The samples were taken randomly from the leaves of three plants of each independent (WT, *Atkai2*, OE1, and OE2) line after 0−, 3−, 6−, and 12 h under cold treatment (4°C). One-way ANOVA was used to analyzed all data, and HSD Tukey’s test was used to perform multiple comparisons at *P < 0.05* significant level (*n* = 3). Bars with uncommon letters showing significant difference at *P < 0.05.* An “h” at the *X*-axis of each graph represents time in hours under cold stress.

Like other abiotic stresses, cold stress can also increase the production of ROS in plants that can cause cellular oxidative damage when over-accumulated in cells (Karuppanapandian and Manoharan, 2008; Mafakheri et al., 2010). H_2_O_2_ is considered as a relatively long-lived molecule and moderately reactive, which can disseminate short distances away from its production site. H_2_O_2_ causes inactivation of enzymes by oxidizing their thiol groups. H_2_O_2_ enables it to diffuse the damage and also act as a messenger in the stress signaling response and thus can travel freely across membranes (Møller et al., 2007). ROS can cause oxidation of membrane lipids and degrade the cell membrane while MDA has been reported as an end product of lipid peroxidation, which is why MDA and H_2_O_2_ levels are markers of determining necrosis and cell damage in living organisms (MaBgorzata and Andrzej, 2016). In order to know whether the involvement of H_2_O_2_ in cold accumulation, we measured H_2_O_2_ content in *SsKAI2* OEs, *Atkai2*, and WT under cold stress. The results showed that cold stress induced a significant increase of H_2_O_2_ content in the WT, while in the *SsKAI2* OEs, the H_2_O_2_ content was not significantly increased in response to the cold stress (Figure 4A). Meanwhile, the results also revealed that the *kai2* mutant had a higher increase of H_2_O_2_ level in comparison with WT after 6 h by cold treatment. An end product of lipid peroxidation, MDA, is a biochemical marker for the measurement of cell epidermal layer degradation. MDA level was increased in *kai2* and wild-type *Arabidopsis* under cold stress, but on the other hand, *SsKAI2* OEs had a decreased level of MDA contents with time under cold stress (Figure 4B). These results demonstrated that the stress-induced accumulation of H_2_O_2_ is strictly regulated by *KAI2*, which further led to enhanced stress tolerance in *Arabidopsis*.

### SsKAI2 OEs Had Enhanced the Level of Enzymatic Anti-oxidants and Glutathione Under Cold Stress

**FIGURE 5:**
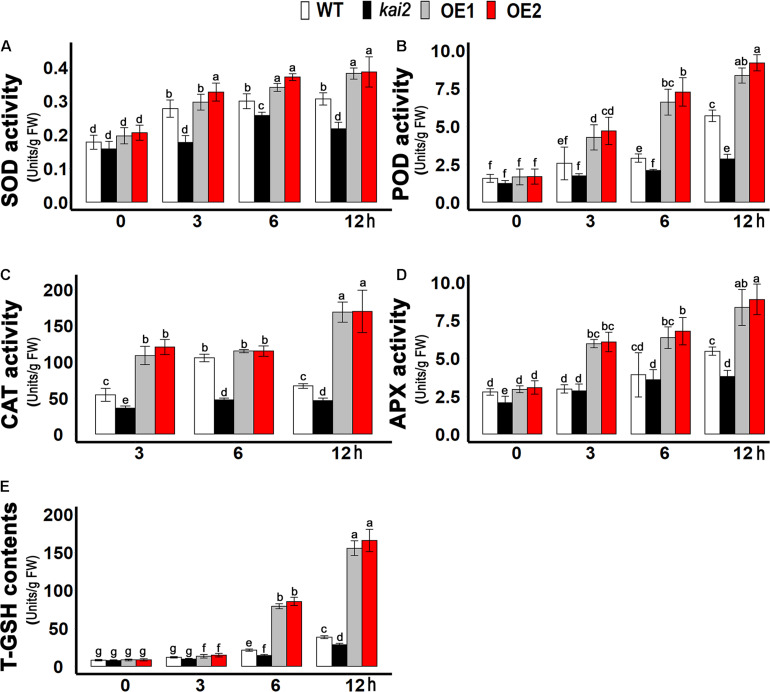
Anti-oxidants contents raised in *SsKAI2* OEs under cold stress. **(A)** SOD. **(B)** POD. **(C)** CAT. **(D)** APX. **(E)** T-GSH. Twenty-five-day-old plants were subjected to cold treatment. The samples were randomly taken from the leaves of three plants of each genotype after 0−, 3−, 6−, and 12 h under cold treatment (4°C). One-way ANOVA was used to analyzed all data, and HSD Tukey’s test was used to perform multiple comparisons at *P < 0.05* significant level (*n* = 3). Bars with uncommon letters showing significant difference at *P < 0.05.*

Ascorbate peroxidase (APX), catalase (CAT), peroxidase (POD), and superoxide dismutase (SOD) are the key enzymatic anti-oxidants which prevent the cell necrosis by scavenging ROS and alleviate oxidative stress. We further investigated the enzymatic anti-oxidants level of different *Arabidopsis* lines under cold stress. In this study, we demonstrated that the SOD activity was significantly higher in *SsKAI2* OEs after six and 12 h of cold stress, but it was much lower in *kai2* mutant in comparison with wild-type and *SsKAI2* OEs during each time point of cold stress (Figure 5A). Nevertheless, under different time points of cold stress, the activity of other anti-oxidant enzymes, such as POD, CAT, and APX, was increased dramatically in *SsKAI2* OEs (Figures 5B–D). Glutathione is a non-enzymatic anti-oxidant in the plant, which protects cellular damage from ROS under environmental stresses (Edwards et al., 2000). The results showed that *SsKAI2* OEs could produce higher concentrations of T-GSH as compared to *kai2* mutant and wild-type plants under cold stress (Figure 5E). These results suggested that *KAI2* conferred cold stress via activating enzymatic and non-enzymatic anti-oxidant systems in *Arabidopsis*.

### SsKAI2 OEs Had Induced Expression Levels of CSPs Genes and CBFs Under Cold Stress

**FIGURE 6:**
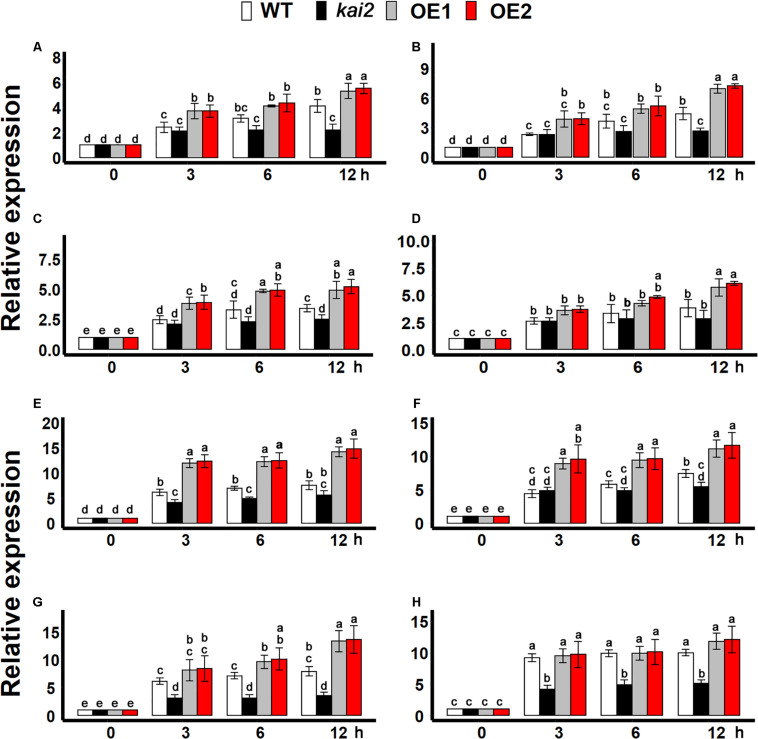
*SsKAI2* OEs have enhanced cold-acclimation-related genes. **(A)** *CSP1*. **(B)** *CSP2*. **(C)** *CSP3*. **(D)** *CSP4*. **(E)** *CBF1.* **(F)** *CBF2.* **(G)** *CBF3.* **(H)** *CBF4.* Expression of cold-acclimation-related genes was investigated in three plants of each line after 0- (control at 22°C), 3−, 6−, and 12 h of cold treatment (4°C). The samples were randomly taken from the leaves of five plants of each treatment. *Arabidopsis thaliana ACTIN 2* was taken as a reference gene, and control treatment at 0 h was considered as 1. One-way ANOVA was used to analyzed all data, and HSD Tukey’s test was used to perform multiple comparisons at *P < 0.05* significant level (*n* = 3). Bars with uncommon letters showing significant difference at *P < 0.05.* An “h” at the *X*-axis of each graph represents time in hours under cold stress.

During cold stress acclimation, cold-shock protein (CSP) genes, and C-repeat binding factors (CBFs) transcription factors are central regulators (Yamaguchishinozaki and Shinozaki, 1994; Chinnusamy et al., 2010). We found that the expression of all CSP genes was more significantly induced by cold treatment in the *SsKAI2* OEs as compared to *kai2* mutant and wild-type *Arabidopsis* (Figure 6A). Although the cold stress could significantly induce the expression of all CBF transcription factors in all the *SsKAI2* OEs, the *SsKAI2* OEs exhibited a much higher expression level than *kai2* mutant and wild-type plants (Figure 6B). These results suggested that the *KAI2* could potentially target the CSPs and CBFs in the regulation of cold acclimation in *Arabidopsis*.

### SsKAI2 OEs Exhibited Hypersensitivity to ABA During Seed Germination and Stomatal Aperture

**FIGURE 7:**
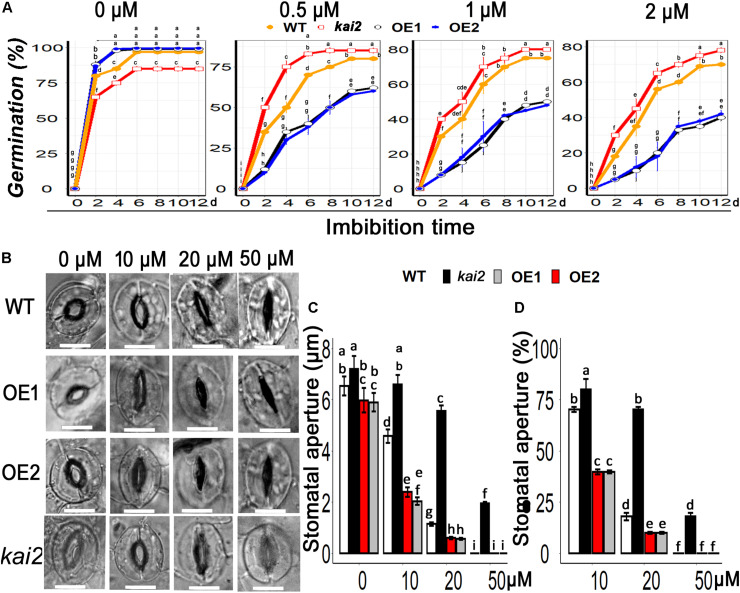
*SsKAI2* OEs were hypersensitive to exogenous ABA. **(A)** An effect of ABA on seed germination in *KAI2* overexpression line1 (OE1), line2 (OE2), WT (Col-0), and *Atkai2 Arabidopsis*. **(B)** *KAI2* plants stomata showed hypersensitivity to ABA. 25-day-old plant leaves were selected for this treatment; white bar = 50 μM. **(C)** Stomatal aperture under different concentrations of ABA. **(D)** Stomatal aperture percentage closed after ABA treatment. Data shown are the mean ± SD of 15 replicates. One-way ANOVA was used to analyzed all data, and HSD Tukey’s test was used to perform multiple comparisons at *P < 0.05* significant level (*n* = 15). Bars or points with uncommon letters showing significant difference at *P < 0.05.*

Abscisic acid is the fundamental phytohormone that positively regulates the abiotic stress adaptation in various plants. To clarify whether karrikins could potentially interact with ABA in the regulation of cold acclimation, firstly we investigated the senstivity of *SsKAI2* to ABA. Then we checked the expression level of ABA biosynthesis, ABA catabolism, and ABA signaling genes. The results showed that the seed germination in the *SsKAI2* OEs was more likely to be inhibited in MS medium supplemented with ABA in comparison with wild-type, while *Atkai2* seeds were less senstive to ABA as compare to wild-type (Figure 7A). The stomata started to close when *SsKAI2* OEs leaves were incubated in ABA supplemented MES (2-(N-morpholino) ethane sulfonic acid) buffer (Figure 7B). Stomatal aperture decreased significantly in *SsKAI2* OEs than wild-type and *kai2* mutant when leaves were dipped in the medium containing 10 or 20 μM ABA. Stomata were completely closed when leaves were dipped in the solution containing 50 μM ABA (Figures 7C,D). These results demonstrated that overexpression of *KAI2* could lead to hypersensitivity to ABA, suggesting a potential interaction between karrikins and ABA.

**FIGURE 8:**
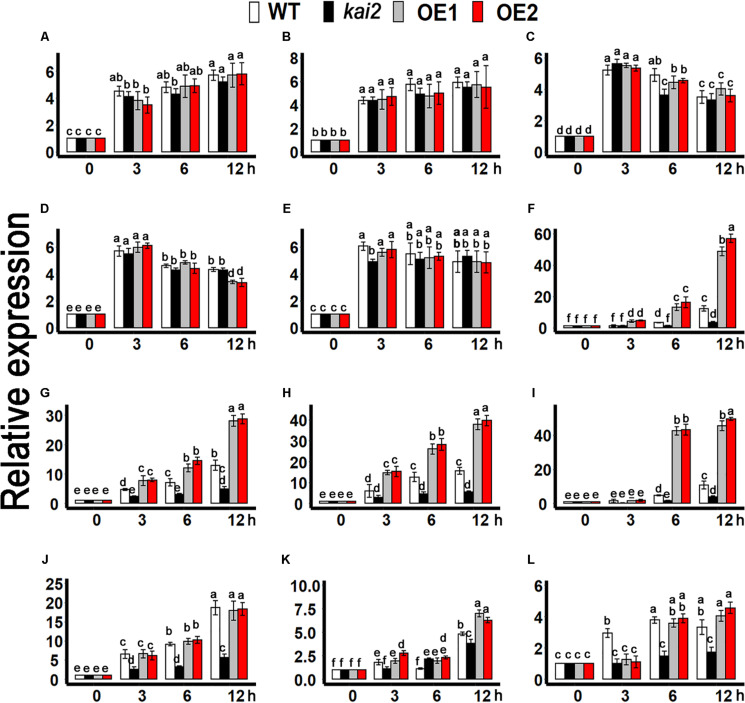
ABA-related genes expression in *SsKAI2* OEs under cold stress. **(A)** *NCED3*. **(B)** *AAO3*. **(C)** *CYP707A1*. **(D)** *CYP707A2*. **(E)** *CYP707A3*. **(F)** *ABI3*. **(G)** *ABI5*. **(H)** *ABF1*. **(I)** *MYB3R2*. **(J)** *MYB96*. **(K)** *SIZ1*. **(L)** *SnRK2.3*. Twenty-five-day-old plants of *SsKAI2* overexpression line1 (OE1), overexpression line1 (OE2), *Atkai2*, and WT (Col-0) *Arabidopsis* were subjected to cold stress (4°C), and samples were taken on given time points. The samples were randomly taken from the aerial of five plants of each treatment. *Arabidopsis thaliana ACTIN2* was taken as a reference gene, control treatment at 0 h was considered as 1. The data shown in the figure are the mean ± SD of three replicates. One-way ANOVA was used to analyzed all data, and HSD Tukey’s test was used to perform multiple comparisons at *P < 0.05* significant level (*n* = 3). Bars with uncommon letters showing significant difference at *P < 0.05.*

Furthermore, to clarify the association of karrikins regulated cold acclimation to ABA, we determined the expression level of cold-responsive ABA biosynthesis genes such as *NINE-CIS-EPOXYCAROTENOID DIOXYGENASE 3* (*NCED3*) and *ABSCISIC ALDEHYDE OXIDASE 3* (*AAO3*)(Qin and Zeevaart, 1999; Seo et al., 2004; Urano et al., 2009), ABA catabolic genes *CYP707A* family (Okamoto et al., 2006; Umezawa et al., 2010), and ABA signaling genes such as *ABI3*,*ABI5*, *ABF1*, *MYB96*, *MYB3R2*, and *SIZ1* (Choi et al., 2000; Lee et al., 2005; Seo et al., 2009; Yuan et al., 2012; Guo et al., 2013; Liu et al., 2013; Dekkers et al., 2016; Skubacz et al., 2016). The results showed that the expression of *NCED3* and *AAO3* was not likely to be induced by cold treatments in the *SsKAI2* OEs (Figures 8A,B). Under cold stress, the expression of *CYP707A1*, *CYP707A2*, *CYP707A3*, *MYB96*, and *SnRK2.3* had no significant differences in all genotypes (Figures 8C,D,E,J,L). Under cold stress, the expression level of cold responsive ABA signaling genes such as *SIZ1*, and *SnRK2.3* was significantly increased in *SsKAI2* OEs. Meanwhile the expression of the key ABA signaling genes *ABI3*, *ABI5*, *MYB3R2*, *ABF1* was also significantly increased in *SsKAI2* OEs as compared to WT (Figures 8F–I,K). These results suggested that *KAI2* potentially affected the ABA downstream signaling, which could contribute to the enhanced cold tolerance in th*e SsKAI2* OEs.

## Discussion

*KARRIKINS INSENSITIVE2 (KAI2)* is a receptor gene for karrikins, which encodes α/β-fold hydrolase, a hydrophobic pocket which may bind to the karrikins (Boyer et al., 2012; Hamiaux et al., 2012; Scaffidi et al., 2012; Li et al., 2013). *KAI2* has been reported to be involved in the regulation of seed germination, hypocotyl development, and photomorphogenesis. Previously, *KAI2* was reported to be involved in the stomatal closure, regulation of cuticle formation, membrane integrity, and anthocyanin biosynthesis, which contributes to plant alleviation of drought stress (Li et al., 2017). It has been reported that the karrikins-*KAI2* signaling system provided stress tolerance by inhibiting germination in *Arabidopsis* under unfavorable conditions (Wang et al., 2018). A few studies have reported the involvement of *KAI2* in the mitigation of environmental stresses such as osmotic and salinity in *Arabidopsis*, but there was not report regarding the role of karrikins-*KAI2* in the regulation of cold stress. Cold resistant plants have induced level of TSS, TSP, and Proline contents (Hellmann, 2012; Keunen et al., 2013; Tarkowski and van den Ende, 2015), which are interlinked with the ROS homeostasis. Higher level of CSPs and CBFs gene expression is one of the fundamental characters of cold resistant plants (Fowler and Thomashow, 2002; Sasaki et al., 2007). Cold tolerance plants have induced expression level of ABA-responsive genes, which lead to stomata closure, and maintain the ROS balance (Thomashow, 1999; Wasilewska et al., 2008; Chinnusamy et al., 2010; Usadel et al., 2010; Hong et al., 2012; Shi et al., 2012; Jurczyk et al., 2019). In this study, we revealed that *SsKAI2* OEs have higher levels of TSS, TSPs and Proline contents, and induced the expression level of CSPs and CBFs. *SsKAI2* OEs were hypersensitive to ABA, and have induced the expression level of ABA-responsive genes, which are important characteristics of a cold resistant plant, and necessary for ROS homeostasis. In this study, we firstly reported that role of *KAI2* in cold stress resistance in *Arabidopsis*, and revealed the biochemical and physiological mechanisms of *KAI2* in the regulation of cold acclimation.

Among a large number of unfavorable conditions, cold (chilling and freezing) stress significantly limits the plant growth and development, and causes losses of the agricultural productivity. Cold resistant plants have developed a defensive system called cold acclimation (Thomashow, 1999; Stockinger et al., 2001; Shi et al., 2014b). Cold acclimation is a highly complicated process that includes an array of physiological, biochemical, and molecular modifications (Chinnusamy et al., 2010; Nakashima et al., 2014; van Buer et al., 2016). We found that overexpression of *SsKAI2* in *Arabidopsis* recovered after cold stress. *SsKAI2* overexpression lines (OEs) had significantly increased levels of proline, total soluble sugars, and total soluble protein. Under cold stress, cold-resistant plants produce an excessive level of soluble sugars, which directly interacted with the phosphate in the lipid headgroups of the cell membrane and decreased the membrane permeability (Strauss and Hauser, 1986). Soluble sugars accumulated in the apoplast of cold-stressed plants also suggested having a role in the protection of the plasma membrane (Valluru et al., 2008). At the same time, cold-resistant plant cells produce the proline, which helps the synthesis of specific proteins necessary for plasma membrane protection (Song et al., 2011; Tian et al., 2011; Melnikov et al., 2016). Furthermore, stress-resistant plants accumulate TSS and TSP to prevent the membrane damage and produce proline, which also plays a unique role in the synthesis of new proteins and may have a role in stress alleviation (Tarkowski and van den Ende, 2015; Li et al., 2018; Ni et al., 2018; Sadiq et al., 2018). In non-resistant plants, cold stress disrupts the cell membrane and cause leakage of electrolytes from the cytosol. Electrolytes leakage could cause the death of the plant (Demidchik et al., 2014). Our results are suggesting that *SsKAI2* accumulated a significant amount of soluble sugars and proteins, which may strengthen the plasma membrane and protected *SsKAI2* OEs from more electrolyte leakage under cold stresses.

Overproduction of ROS in plants under various abiotic stresses, including cold stress, causes oxidative cellular damage (Karuppanapandian and Manoharan, 2008; Mafakheri et al., 2010; van Buer et al., 2016). Among ROS, H_2_O_2_ is a relatively long-lived molecule and moderately reactive, disseminating short distances away from its production site. H_2_O_2_ enables it to diffuse the damage, act as a messenger in the stress signaling response, and travel freely across membranes (Møller et al., 2007). H_2_O_2_ can cause oxidation of membrane lipids and degrade the cell membrane, while MDA has been reported as an end product of lipid peroxidation, which is why MDA and H_2_O_2_ levels are markers of determining necrosis and cell damage in living organisms (MaBgorzata and Andrzej, 2016). In this study, we found that *SsKAI2* OEs produced significantly lower amount of H_2_O_2_, and MDA level than WT under cold stress (Figures 3A,B). *SsKAI2* had lower percentage of EL than WT under freezing temperature (Figure 2C), these results are consistent with previous report, demonstrating that a cold-sensitive *S. lycopersicum* genotype under cold stress produced significantly higher MDA and H_2_O_2_ content compared with controls. Han et al. (2017) found an increased level of MDA and EL contents in rice seedlings under cold stress. Similarly, Xue et al. (2019) reported that WT plants accumulate higher levels of H_2_O_2_ compared with transgenic *Ammopiptanthus mongolicus* under cold stress. These results are suggesting that *SsKAI2* provided shield to cold stress via reducing H_2_O_2_ level, decreasing MDA content, and protecting plant cells from electrolyte leakage.

Various anti-oxidative defense systems scavenge ROS under steady-state conditions (Navrot et al., 2007). In anti-oxidative defense systems, ascorbate peroxidase (APX), catalase (CAT), peroxidase (POD), and superoxide dismutase (SOD) are the key enzymatic anti-oxidants that prevent cell necrosis by scavenging ROS and alleviate oxidative stress (Sairam et al., 2005; Fabio et al., 2007; Songbi and Bruria, 2013; Luis et al., 2018). When we investigated the activity of different enzymatic anti-oxidants such as APX, SOD, POD, and CAT in different *Arabidopsis* lines under cold stress, we found that the SOD activity was significantly higher in *SsKAI2* OEs after six and 12 h of cold stress, but it was much lower in *Atkai2* mutant than wild-type at each time point after cold stress (Figure 5A). Results here are agreement with a previous report, describing that cucumber seedling showed an induction in SOD activity under the cold stress (Zhao et al., 2016). Under different time points of cold stress, the activity of other anti-oxidant enzymes, such as POD, CAT, and APX, was increased dramatically in *SsKAI2* OEs as compared to WT (Figures 5B–D). Previous studies showed an increased CAT activity in *Cynodon dactylon*, *Capsella bursa pastoris*, and *Citrus reticulata*, under cold stress (Shi et al., 2014a; Wani et al., 2018; Mohammadrezakhani et al., 2019). A higher activity of APX was detected in cold tolerant *Jatropha macrocarpa*, whereas reduction in APX activity was observed in cold sensitive *Jatropha macrocarpa* (Spano et al., 2017). Glutathione is a non-enzymatic anti-oxidant in the plant, which protects cellular damage from ROS under environmental stresses (Edwards et al., 2000). Cheng et al. (2016) observed the significantly higher GSH level in treated *Citrullus lanatus* compared with control samples under cold stress 24 h after treatment. Similarly, Wang Q. J. et al. (2016) demonstrated the increased GSH levels in transgenic apple seedlings as compared with WT under low temperature stress. We found that *SsKAI2* OEs produced higher concentrations of T-GSH, while *Atkai2* produced a significantly lower T-GSH contents than the WT plant under cold stress (Figure 5E). These results suggested that *KAI2* conferred cold stress via activating enzymatic and non-enzymatic anti-oxidant systems in *Arabidopsis*.

During the process of cold acclimation, *COLD SHOCK PROTEINS* (CSPs) and *C-REPEAT BINDING FACTORS* (CBFs) were highly expressed in the cold-resistant plants. In the model plants *Arabidopsis* and poplar, the expression level of four CSP genes is differentially regulated in response to cold cues (Karlson and Imai, 2003; Benedict et al., 2006; Sasaki et al., 2007; Nakaminami et al., 2009). Overexpression of AtCBFs in the other plant species, or overexpression of CBFs from other species in *Arabidopsis* alleviated the freezing tolerance (Benedict et al., 2006; Tondelli et al., 2011). Previous studies revealed that the exogenous application with karrikins in *Arabidopsis* up-regulated the expression level of *COLD SHOCK PROTEIN 2* (Baldrianová et al., 2015), which might be a reason for induction in cold resistance in *Arabidopsis* by the exogenous application of KAR_1_ (Supplementary Figure 2). It has also been reported that cold shock proteins were up-regulated by the transcription factors *C-REPEAT BINDING FACTORS* (CBFs) in response to cold stress (Fowler and Thomashow, 2002; Gilmour et al., 2004). In this study, *SsKAI2* OEs has the highest level of CSPs and CBFs genes expression, while *kai2* mutant exhibited the lowest expression level when compared to wild-type plants under cold stress (Figure 6). These results are consist with the previous studies showing that the cold resistant plants had a higher expression level of CSPs and CBFs (Fowler and Thomashow, 2002; Sasaki et al., 2007), suggesting that *KAI2* might have a relationship with CSPs and CBFs in the regulation of cold acclimation in *Arabidopsis*.

Cold stress, same as other abiotic stresses, also cause water imbalance in plants and increase the abscisic acid (ABA) biosynthesis which could trigger the stomatal closure. Hence, stomatal closure is an adaptive strategy to drought, and cold (Shi and Yang, 2014; Jurczyk et al., 2019). In this study, *SsKAI2* OEs were hypersensitive to ABA, and exogenous application of ABA, severely repressed seed germination, and caused induction in stomatal closure whereas *Atkai2* mutants were not that sensitive to ABA as compared to WT (Figure 7), which are in agreement with the results of Li et al. (2017). However, it has also been suggested that ABA played a role in cold acclimation via triggering the expression of a set of stress responsive genes (Shi and Yang, 2014). ABA causes several changes in plant molecular, developmental, and physiological progressions resulting in plant adaptation to environmental stresses (Ton et al., 2009). Our results are consistent with Lee and Luan (2012), depicted that abiotic stresses stimulate ABA production which further triggers the expression of stomatal closure and stress-related genes. We found that *SsKAI2* OEs promoted the expression level of ABA responsive genes, such as *ABI3, ABI5, MYB96, MYB3R2*, and *ABF1* under cold stress (Figure 8), which is an agreement with the results of Li et al. (2017) suggesting that *KAI2* might regulate the abiotic stress tolerance could be ABA-dependent. The relationship of ABA with redox homeostasis is well documented in different studies (Wang H. et al., 2016; Postiglione and Muday, 2020; Wenjing et al., 2020). Altogether, our results are suggesting that *SsKAI2* enhanced response to ABA and induced expression level of ABA-responsive genes might be a pathway leading to redox homeostasis under cold stress.

Conclusively, in this study, the karrikins receptor gene *KAI2* from the perennial woody plant *Sapium sebiferum* was the first time isolated and characterized under cold stress. The results of this report represented a novel function of *KAI2* in the regulation of cold stress resistance in *Arabidopsis* by maintaining the redox homeostasis, increasing the ABA sensitivity, and inducing the expression of CSPs or CBFs genes. This study is providing foundations for researchers to explore abiotic stresses regulation functions of *KAI2* in different plant species. Our discovery provides a foundation for the production of cold resistant plants. This study is beneficial for improving agronomic, horticultural, and forest plant research.

## Data Availability Statement

The original contributions presented in the study are included in the article/Supplementary Material, further inquiries can be directed to the corresponding author.

## Author Contributions

FS, JN, and LW designed the experiments. JN, FS, YY, HH, and RW carried out the experiments. JN, FS, and YY analyzed the data and took photographs. FS wrote the manuscript. All authors read and approved the final manuscript.

## Conflict of Interest

The authors declare that the research was conducted in the absence of any commercial or financial relationships that could be construed as a potential conflict of interest.

## Footnotes

1 https://www.ncbi.nlm.nih.gov/sra/SRX656554

## Supplementary Material

Supplementary Figure 1 Bioinformatics analysis of *KAI2*.

Supplementary Figure 2 Exogenous application of KAR_1_ alleviated cold tolerance in *Arabidopsis.*

Supplementary Table 1 List of primers.

Supplementary Data Sheet 1 Proteins sequences and the list of genes used in the manuscript.

## Abbreviations

ABA: abscisic acid
APX: ascorbate peroxidase
CAT: catalase
CBFs: C-REPEAT BINDING FACTORS
CSPs: cold shock proteins
H_2_O_2_: hydrogen peroxide
*KAI2*: *KARRIKINS INSENSITIVE2*
KAR_1_: karrikin 1
KAR_2_: karrikin 2
KCl: potassium chloride
MDA: malondialdehyde
MES: 2-(N-morpholino) ethanesulfonic acid
POD: peroxidase
ROS: reactive oxygen species
SOD: superoxide dismutase
TB: toluidine blue
T-GSH: total glutathione
TSP: total soluble protein
TSS: total soluble sugars.
