# Supplementary material for: Overexpression of Karrikins Receptor Gene Sapium sebiferum KAI2 Promotes the Cold Stress Tolerance via Regulating the Redox Homeostasis in Arabidopsis thaliana
Source: Front Plant Sci. 2021 Jul 15;12:657960. doi: 10.3389/fpls.2021.657960 (PMC8320022; doi:10.3389/fpls.2021.657960)
Supplement: Supplementary Figure 1 — Bioinformatics analysis of KAI2. [file Image_1.pdf]

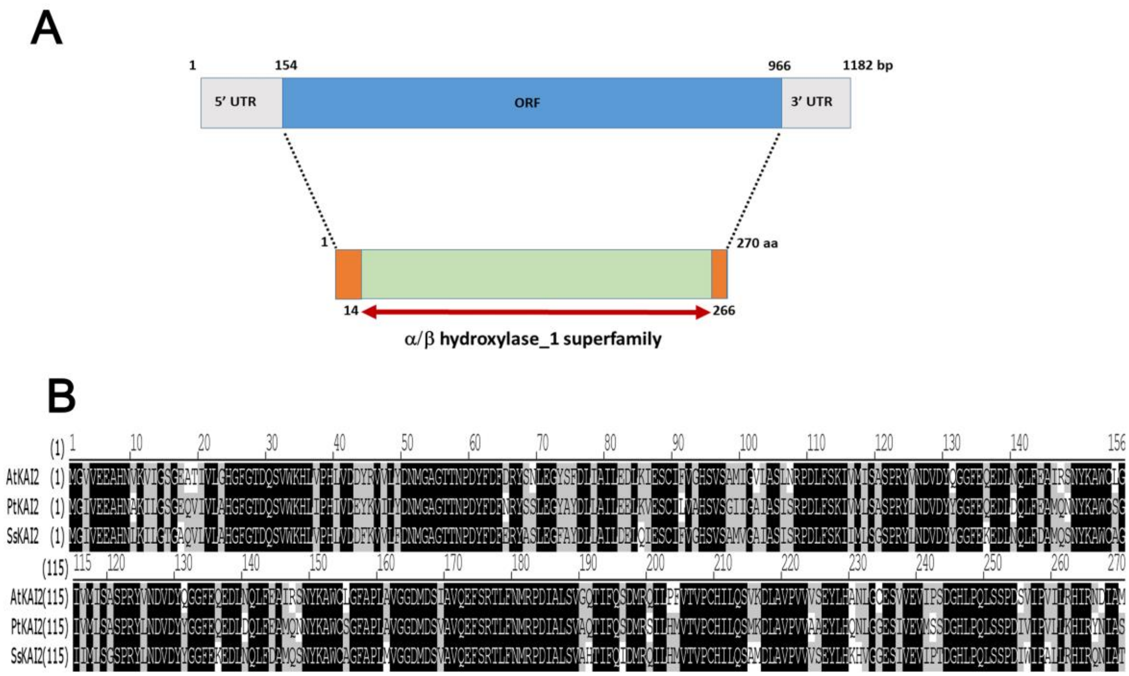

Bio-informatics analysis of *SsKAI2*.

A, Schematic representation of *Sapium sebiferum KAI2*. The *SsKAI2* gene is 1,182 bp with a 153-bp 5' untranslated region, a 813-bp coding region, a 216-bp 3' untranslated region, and no introns. *SsKAI2* translated to make 270 amino acid proteins, which has α/β hydroxylase domain. **B**, *Sapium sebiferum* KAI2 protein alignment with *Arabidopsis* and *Populus* KAI2 protein. The black background indicates similar and grey background shows a conserved sequence.
