# Supplementary material for: Overexpression of Karrikins Receptor Gene Sapium sebiferum KAI2 Promotes the Cold Stress Tolerance via Regulating the Redox Homeostasis in Arabidopsis thaliana
Source: Front Plant Sci. 2021 Jul 15;12:657960. doi: 10.3389/fpls.2021.657960 (PMC8320022; doi:10.3389/fpls.2021.657960)
Supplement: Supplementary Figure 2 — Exogenous application of KAR1 alleviated cold tolerance in Arabidopsis. [file Image_2.pdf]

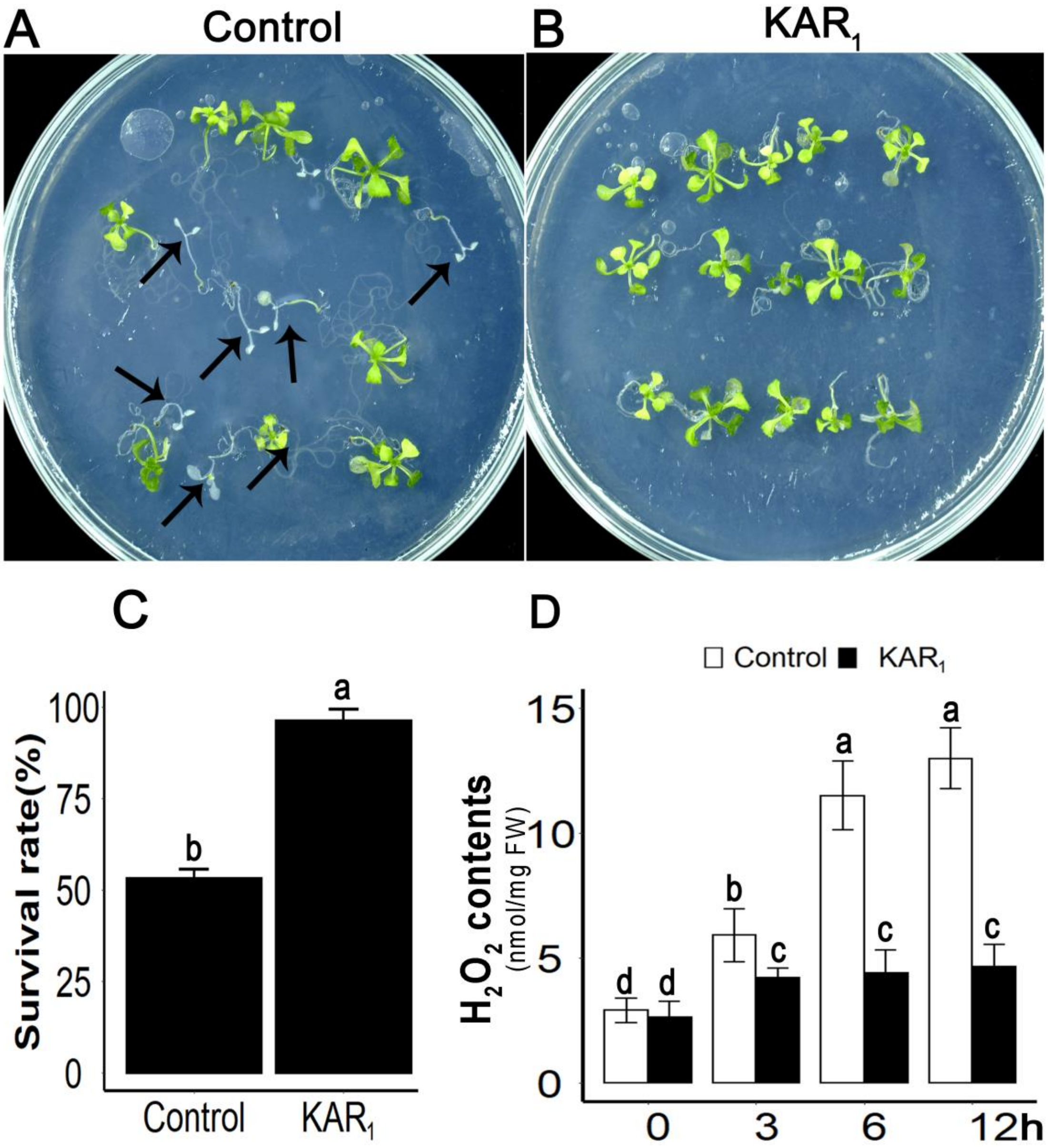

Supplementary Figure 2 Exogenous application of KAR_1 alleviated cold stress tolerance in *Arabidopsis*. **A**, Control (½MS medium). **B**, KAR_1 (½MS medium containing 1 μl KAR_1). **C**, statistical analysis of survival rate under cold stress. **D**, H_2O_2 contents under cold stress. The seeds of the *Arabidopsis* Columbia-0 (Col-0) were surfaces sterilized with 70% (v/v) ethanol for 2 minutes, then incubated in 10% (v/v) sodium hypochlorite (NaClO) for 10 minutes at room temperature, and washed thrice with double distilled water. The sterilized seeds were plated on Control (without KAR_1), and KAR_1 (1 ml) supplemented ½ Murashige and Skoog (MS) medium containing 1%

(w/v) sucrose and 0.8% (w/v) agar and placed at 4 degrees Celsius (℃) for two days. Seeds were germinated in a growth room 16/8 h (day/night) photoperiod at 22 ℃. For phenotypical analysis under cold stress, five-day-old *Arabidopsis* seedlings were cold acclimatized to 4 ℃ for 12 hours and then subjected to cold treatment at -20 ℃ for an hour. The plants were again kept at 4 ℃ for 12 hours, the plants were then shifted to a plant growth room with a 16/8 h photoperiod at 22 ℃, approximately 120 µmol photons/m^2/s, and 75% humidity. The recovery rate was measured ten days after the cold-shock treatment. Photographs were taken by a Nikon D90 having Nikon DX AF-S NIKKOR 18-105 mm lens (Nikon Corporation, Tokyo, Japan). For H_2O_2 analysis, the samples were randomly taken from the leaves of five plants of each treatment after 0 (control at 22 ℃), 3-, 6-, and 12 hours of cold treatment (4 ℃). Samples were immediately frozen in liquid nitrogen, the stored at -80 ℃. The hydrogen peroxide (H_2O_2), was analysed by an H_2O_2 assay kit (Nanjing Jiancheng Bioengineering Institute, Nanjing, China), respectively, as previously described by Ni et al. (2018). One-way ANOVA was used to analysed all data, and HSD Tukey's test was used to perform multiple comparisons at $P<0.05$ significant level (n = 5). Bars with uncommon letters showing significant difference at $P<0.05$. "h" represents the duration (in hours) under cold stress.
