# Supplementary material for: Overexpression of Karrikins Receptor Gene Sapium sebiferum KAI2 Promotes the Cold Stress Tolerance via Regulating the Redox Homeostasis in Arabidopsis thaliana
Source: Front Plant Sci. 2021 Jul 15;12:657960. doi: 10.3389/fpls.2021.657960 (PMC8320022; doi:10.3389/fpls.2021.657960)
Supplement: Supplementary Table 1 — List of primers. [file Table_1.docx]

**Supplementary Table 1 List of primers**

| **Gene Name** | **Primer Type** | **Sequence(5'------3' end)** |
| --- | --- | --- |
| *SsKAI2* | Sense Primer | ATAGTCACACAATTTTACTCCATTAGCTTG |
| *Full length* | Anti-sense Primer | CCACAATAACATTAGCAGCCAAAAGAAAGT |
| *SsKAI2 (qRT-PCR)* | Sense Primer | CTATTGCTTCCATTTCTCGCCCAGATC |
|  | Anti-sense Primer | TCTCAAATCCTCCATAGTAATCCACATCGT |
| *AtNCED3* | Sense Primer | ATGGCTTGGTGGCAATCATACTCAG |
|  | Anti-sense Primer | GGCTTAACAACAATGGCGGGAGA |
| *AtAAO3* | Sense Primer | GTAGTTGAGGTTGGAGGAATTGAGATAGG |
|  | Anti-sense Primer | AACTGCTCTCGGATGTCGTGCTA |
| *AtCYP707A1* | Sense Primer | AAGCTCGGAAGGAACTCTCACAGAT |
|  | Anti-sense Primer | CGTGTCTCTAGCGGCGAAGATTAC |
| *AtCYP707A2* | Sense Primer | CCGCTTCTGTCTTAACTTGGCTTCT |
|  | Anti-sense Primer | CTTGCTGCTCTTAGTGTCTCTTGTATCA |
| *AtCYP707A3* | Sense Primer | AGCCATCTTCTTCCATCAAGGAGATTATC |
|  | Anti-sense Primer | TTCCTGGTAAGTGTTGAGTTGAGTTCC |
| *AtCBF1* | Sense Primer | CTGGACATGGAGGAGACGATGGT |
|  | Anti-sense Primer | TTGAACAGACGGCGGCGGTA |
| *AtCBF2* | Sense Primer | GAATCAACCTGTGCCAAG |
|  | Anti-sense Primer | CATCGCCTCTTCATCCAT |
| *AtCBF3* | Sense Primer | CGGTAAGTGGGTTTGTGAGGTTAGAG |
|  | Anti-sense Primer | TCCAAGCCGAGTCAGCGAAATTG |
| *AtCBF4* | Sense Primer | GGGTTTGTGAAGTTAGAGAGCCTAATAAGA |
|  | Anti-sense Primer | ATACGAAGCCGCCAAGCAGAATC |
| *SsUBQ10* | Sense Primer | GTATCGTGTTGGATTCTGGTGATGGT |
|  | Anti-sense Primer | CGGCAGTGGTGGTGAAGGAGTA |
| *AtABI3* | Sense Primer | TGAATCCGTACCAATATCCTTATGTTCCTG |
|  | Anti-sense Primer | AACCTCCTCTGTCTCGCCATCC |
| *AtABI5* | Sense Primer | GTAGTAGTAATGGACAGAACAATGCTCAGA |
|  | Anti-sense Primer | ACCACACCAGCCTTCACCAAGA |
| *AtABF1* | Sense Primer | CAACAACTTAGGCGGCGATACTTCT |
|  | Anti-sense Primer | CGGCTCACCTAATGTGCTCTGAAG |
| *AtMYB3R2* | Sense Primer | GCACAAAGAGGAAACGGCAACAAAT |
|  | Anti-sense Primer | ACCATAGACCACACCAGAGAAGAGG |
| *AtCSP1* | Sense Primer | TGTAGCACGCATTATCATTACCTCCTC |
|  | Anti-sense Primer | TGTTCTGAAGGCGGTGGAGGTT |
| *AtCSP2* | Sense Primer | GGTGCAATCCCTGGCGAAATGT |
|  | Anti-sense Primer | TGGCGGCGAAGGTGAATGTTAC |
| *AtCSP3* | Sense Primer | TCCACCAGAACCACCACGACAA |
|  | Anti-sense Primer | ACAGCGGTGGTGGTGGTTCA |
| *AtCSP4* | Sense Primer | ACAGCGGTGGTGGTGGTTCA |
|  | Anti-sense Primer | TCTCGCCATGTGACCTGGTTCA |
| *AtMYB96* | Sense Primer | GACCGTTGTTATGATCCATCCTCTTCTT |
|  | Anti-sense Primer | TGTTCTCAGCACTTGACGCATATACC |
| *AtSIZ1* | Sense Primer | CACTAGCCATCGGCATTCTAACTGTT |
|  | Anti-sense Primer | GTTGAGTCTGAATCCACATCTTCCATCT |
| *AtSnRK2.3* | Sense Primer | GGAGATCATTAACCACAGGTCACTAAGG |
|  | Anti-sense Primer | GTTCACCGCCAGAAGCATATTCCA |
| *AtACTIN2* | Sense Primer | GTATTGTGCTGGATTCTGGTGATGGT |
|  | Anti-sense Primer | CGGAGGATGGCATGAGGAAGAGA |
