# Supplementary material for: Overexpression of Karrikins Receptor Gene Sapium sebiferum KAI2 Promotes the Cold Stress Tolerance via Regulating the Redox Homeostasis in Arabidopsis thaliana
Source: Front Plant Sci. 2021 Jul 15;12:657960. doi: 10.3389/fpls.2021.657960 (PMC8320022; doi:10.3389/fpls.2021.657960)
Supplement: Supplementary Data Sheet 1 — Proteins sequences and the list of genes used in the manuscript. [file Data_Sheet_1.docx]

**Sequences of proteins used in phylogenetic analysis**

**SsKAI2**

AATGTAATATCTGGCCTCATATTGAAAAGAGTTCGGCTAAATTCTCTAACCGCCGCCGGCMGIVEEAHNLKILGTGAQVLVLAHGFGTDQSVWKHLVPHLVDDFKVVLFDNMGAGTTNPDYFDFERYASLEGFAYDLLAILDELQIESCIFVGHSVSAMVGAIASISRPDLFSKIIMLSGSPRYLNDVDYYGGFEKEDLNQLFDAMQSNYKAWCAGFAPLMVGGDMDSVAVQEFSRTLFNMRPDIALSVAHTIFQIDMRQILHMVTVPCHILQSAMDLAVPVVSEYLHKHVGGESIVEVIPTDGHLPQLSS

**AhKAI2**

MGIVEEAHNVKVLGSGDKFIILAHGFGTDQSVWKHFVTHLLDDYRVILYDNMGAGTTNPDYFDFERHSLEGYAYDLLAILEELSVHSCIFVGHSVSAMIGVVASIARPDLFSKLIMVSASPRYLNDVNYYGGFEQEDLDQLFDAMSANYKAWCNGFAPLAVGGDMESVAVQEFSRTLFNMRPDIALVVSRTIFQSDMRQILVHVTVPCHILQSKKDMAVPVVVSEYLHQNLGGISVVEVMATDGHLPQLSSPDIVIPVLLKHIQHDIVV

**ApKAI2**

MGIVEEAHNVRVVGKGNQIVILAHGFGTDQSVWKHMVPHLVDDYRVILYDNMGAGTTNPDYFDFERYSTIEGFVYDLLSILEELQVQSCIFVGHSLSAMVGLLASISHPHLFTKLILISASPRFLNDPEYFGGFQQEDLSQLYDGIRSNYKAWCSGFAPLVIGGDMESVAVQEFSRTLFNMRPDIALSLAQTIFQFDMRPILCHVTVPCHIIQSTKDLAAPVVVAEYLQRNIGGKTIVEVMPTEGHLPQLSSPDIVIPVLLNHIRYDLTAPK

**AtKAI2**

MGVVEEAHNVKVIGSGEATIVLGHGFGTDQSVWKHLVPHLVDDYRVVLYDNMGAGTTNPDYFDFDRYSNLEGYSFDLIAILEDLKIESCIFVGHSVSAMIGVLASLNRPDLFSKIVMISASPRYVNDVDYQGGFEQEDLNQLFEAIRSNYKAWCLGFAPLAGGDMDSIAVQEFSRTLFNMRPDIALSVGQTIFQSDMRQILPFVTVPCHILQSVKDLAVPVVVSEYLHANLGCESVVEVIPSDGHLPQLSSPDSVIPVILRHIRNDIAM

**BnKAI2**

MGVVEEAHNVKVIGSGNQGTIVLGHGFGTDQSVWKHLVPHLVEDYRLVLYDNMGAGTTNPEYFDFDRYSTLEGFSFDLIAILEDLQIESCIFVGHSLSGMVGVLASLNRPDLFSKIVMISASPRYVNDADYQGGFEEEDLNQLFEAMRSNYKAWCLGFAPLAVGGDLDSVAVQEFSRXLFNMRPDIALSLAQTIFHSDMRQILPFVSVPCHIVQSVKDLAVPVAVSEYLHNNLGSESVVEVMSSDGHLPQLSSPDSVIPVLLRHIRNDIAL

**BrKAI2**

MGVVEEAHNVKVIGTGTQATIVLGHGFGTDQSVWKHLVPHLLEDYRIVLYDNMGAGTTNPDYFDFDRYSTLQGYSFDLIAILEDLHIESCIFVGHSVSAMVGLLASLNRPDLFSKIVMISASPRYVNDVDYQGGFEQDDLNQLFEAMGSNYKAWCLGFAPLAVGGDMDSVAVQEFSRTLFNMRPDIALSVAQTIFQSDMRQILPFVAVPCHILQSVKDLAVPVAVSEYLHTNLGCESVVEVIPSDGHLPQLSSPDSVIPVLLRHIRNDIAV

**CaKAI2**

MGIVGEAHNVRVLGAGEKTIVLGHGFGTDQSVWKHLVPYLVNEYRVVLYDNMGAGSTNPEYFDFDRYSSLEGYAYDLIAILEELQINCCVYLGHSLSSMTGVVASIFRPDLFSKLILVSASPRFINTDDYYGGFESEEIEQLCQAMESNYKAWISGFAPLVVSGDMDSVAVQEFSRTLFNMRPDIALSVFRTVFTFDLRHFLSRVTVPCHIIQSSMDMSLPVSVSEYLHRNLGGKSIVEIMSTEGHLPHLSAPEATIPVLLRHIAHDITADGDA

**CcKAI2**

MGIVEEAHNVKVLGSGKRCIVLAHGFGTDQSVWKHLVPHLLDEFRVIVYDNMGAGTTNPDYFDFERYSTLDGYAYDLLAILEELRVDSCIFVGHSVSAMIGTVASVSRPDLFTKIIMISASPRYLNDTEYFGGFEHEDLDQLFNAMAANYKAWCSGFAPMAVGGDMDSVAVQEFSRTLFNMRPDIALSVLQTIFHSDMRQILNLVTVPCHIIQSMKDLAVPVVVAEYLHQHLRSHSVVEVMPTEGHLPQLSSPDIVIPVILKHICLDITSKH

**CpKAI2**

MGIVEEAHNVRVLGSGEQVIVLAHGFGTDQSVWRHLVPHLVDDHRVILYDNMGAGTTNPDYFDFGRYATLEGFAYDLLAILEDQQVESCIFVGHSVSAMVGAIASITRPDLFSKIVMISASPRYLNDVDYLGGFDQEDLDQLFEAMRENYKAWCSGFAPLAVGGDMDSLAVQEFSRTLFNMRPDIALSVGQTIFQSDMRQILGLVTVPCHIVQSVKDLAVPVVVSEYLHQNLGGDSIVEVMSSDGHLPQLSSPDIVIPVLLRHIQHDIAA

**CsKAI2**

MGVVEEAHNVKVIGSGNQATIVLGHGFGTDQSVWKHLVPHLVDDYRVVLYDNMGAGTTNPEYFDFDRYSTLEGFSFDLIAILEDLQIESCIFVGHSVSAMVGVLASLNRPDLFSKIVMISASPRYVNAVDYQGGFEQDDLNQLFEAMRSNYKAWCSGFAPLAVGGDLDSIAVQEFSRTLFNMRPDIALSLGQTIFQSDMRHILPFVSVPCHILQSVRDLAVPVSVSEYLHTNLGSESVVEVIPSDGHLPQLSSPDSVVPVVLRHIRNDIDL

**DcKAI2**

MNSRLIGNGEITLVLSHGYGASQAVWDEVVPKLSKRYQLFLFDWCFSEAVKPPLTFDSSKDFSFEGFADELISLLDNYKLKCVVFVGHSMAGMVGCLASIKRPDLFSHLVLVGASPRYVNSDDYQGGFQLTDVENILTNIQTNFQAWAEEFAPMASGITDQIHLKKLIDSFKGMNPTAALPLAKMIFLSDQRHVLEHVKLPCTIIQGTEDIVVPVVVGDYMKCKLKGETSLVNIDKNAHFPQITSPKKFVEILRKILK

**EgKAI2**

MGIVEDAHNVKVLGAGERTIVLAHGFGTDQSVWKHLVPHLVDTHRVVIYDNMGAGTTNPEYFDFERYTLEGYAYDLLAILEELHIESCIFVGHSVSAMVGSIASISRPDLFAKIVMLSGSPRYVNDPDYFGGFEQEDLDQLFEAMRENYKAWCSGFAPLAVGGDMDSVAVQEFSRTLFNMRPDIALSVAQTIFNSDMRSILRLVTVPCHIIQSMKDLAVPVVVSEYLHQNLGGDSIVEVMQSDGHLPQLSSPDTVIPVILRHIRYDIAT

**EsKAI2**

MGVVEEAHNVKVIGSGNQATIVLGHGFGTDQSVWKHLVPHLVDDYRIVLYDNMGAGTTNPDYFDFDRYSTLEGFSFDLIAILEDLQIESCIFVGHSVSAMVGVLASLNRPDLFSKIVMISASPRYVNDVDYQGGFEQDDLNQLFEAMRSNYKAWCLGFAPLAVGGDLDSVAVQEFSRTLFNMRPDIALSVAQTIFQSDMRQILPFVAVPCHILQSVKDLAVPVVVSEYLHTNLGSESVVEVIPSDGHLPQLSSPDSVIPVLLRHIRNDIAV

**FvKAI2**

MGIVEEAHNVRVLGSGQRIIVLAHGFGTDQSVWKHLVPHLVDDYKVLMYDNMGAGTTNPDYFDFDRYSTLEGYAYDLLAILEEFQVESCIFVGHSVSGMIGAIAAVMRPDLFTKLVMIAASPRYLNDSDYYGGFEQEDLEQLFEAIRSNYKAWCSGFAPLAVGGDMDSVAVQEFSRTLFNMRPDIALSVAQTIFQSDMRQVIGMVTVPCHILQSVKDLAVPVVVTEYLHQNLGGESIVEVMSSDGHLPQLSSPDIVIPVLLRHIRHDIAV

**GhKAI2**

MGVVEEAHNLKVLGSGDRVIVLAHGFGTDQSVWKHLVPHLVDDFRVVLYDNMGAGTTNPEYFDFNRYATLEGYAYDLLAILEEIHVDSCIFVGHSVSAMVGAIASISRPDFFSKIIMISGSPRYLNDVDYYGGFEQEELDQLFEAMEANYKAWCSGFAPLVVGGDLESVAVQEFSRTLFNMRPDISLSVAQTIFQSDMRQILNLVTVPCHILQSVKDLAVPVVVSEYLYQNLGGESIVEVMSSDGHLPQLSSPDIVIPVLLKHIRYDISVA

**GmKAI2**

MGIAAEAHNVKILGSGTEYIVLAHGFGTDQSVWKHFVPYLVDNFRVILYDNMGAGTTNPDYFDFERHSILEGYASDLLAILEELQVESCIFVGHSVSAMIGAIASISRPDLFTKLIMVGASPRYLNDVEYYGGFEQEDLDQLFDAMAANYKAWCYGFAPLAVGGDMESVAVQEFSRTLFNMRPDIALIVSRTIFQSDMRQILSLVSVPCHIIQAEKDMAVPMMISEYLHQHIGAESIVEVMATDGHLPQLSSPDTVIPVLLKHIQLDIEARR

**GsKAI2**

MGIAAEAHNVKILGPGTECIVLAHGFGTDQSVWKHLVPYLVDDYRVILYDNMGAGTTNPDYFDFERHSSLEGYASDLLAILEELQVESCIFVGHSVSAMIGAIASISRPDLFTKLIMVSASPRYLNDVEYYGGFEQEDLNQLFDAMAANYKAWCYGFAPLAVGGDMESVAVQEFSRTLFNMRPDIALIVSRTIFQSDMRQILSLVSVPCHIIQAEKDMAVPVMISEYLHQHIGAESIVEVMATDGHLPQLSSPDIVIPVLLKHIQLDIEARR

**HaKAI2**

MGVLEKAHNIKILGSGSQTIILAHGFGTDQSVWKHLVPHLVDDYKVVLFDTMGAGTTNPEYFDFDRYATLEGFAYDVIGILEELRVCSCIYVGHSVSTMIGALASILRPDLFSKLVMIAGSPRYLNDPDYFGGFEQEDLDQLFEAMQSNYKAWCSGFAPLAIGGDMESVAVQEFSRTLFNMRPDIAYSVLQTIFQIDMRNLLRHVTVPCHIIQSMKDLAVPVVVAEYLHQNLGGESIVEVMSTDGHLPQLSSPDVVVPVILRHIRCDIAL

**JcKAI2**

MGIVEEAHNVKIIGAGEQVLVLAHGFGTDQSVWKHLVPHLLDDFKVILYDNMGAGTTNPDYFDFDRYSTLEGYAYDLLAILEELQVESCIFVGHSVSAMIGAIASISRPDLFSKLVMISGSPRYLNDVDYYGGFEQEDLNQLFEAMESNYKAWCSGFAPLAVGGDMDSVAVQEFSRTLFNMRPDIALSVARTIFQSDMRQILHLITVPCHIVQSGKDLAVPVVVSEYLHQNLGSESIVEVMSSDGHLPQLSSPDIVIPVLLRHIRYDIAA

**JrKAI2**

MGIVEDAHNVKVLGFGDRNIVLAHGFGTDQSVWKHLIPHLLDDNKRIILYDNMGAGTTNPDYFDFQRYSTLEGYTYDLLAILEELRVDSCVFVGHSVSAMIGAIASISRPDLFSKIIMVGASPRYLNDVDYYGGFEQEDLDQLFEAMRSNYKAWCSGFAPLAVGGDMDSVAVQEFSRTLFNMRPDIALSVAQTIFQSDMRQILCLVTVPCHIIQSMQDLAVPVVVSEYLHQNLGGESIVEVMSSDGHLPQLSSPEVVIPVLLRHIRHDIAL

**MdKAI2**

MGIVEEAHNVRVVGSGQQVLVLGHGFGTDQSVWKHLVPHLVDDYRVILYDNMGAGTTNPEYFDFERYSTLEGYAYDLLAILEVLHVQSCIFVDHSMSAMIGAIAAVTRPDLFTKLIMIAASPRFLNDVDYYGGFEQEDVEQLFEAIQSNYKAWCSGFASLVVGRDMDSVAIQEFSRTLFNMRPDISLSVAQTIFQSDIRQILRLITVPCHILQSVKDLAVPMVVTEYLHQNLGGKSIVEVMSSDGYLPQLSSPDIVNLVLLKHIRYDIMA

**MnKAI2**

GATAGATAGATAGATAGATAGAGAATCTCTTTGCTCTAAAGGCACATCAAAGTGGCGGTAMGIVSEAHNVKVLGGGNNNNNNNNQTIVLGHGFGTDQSVWKHLIPHLVDEFQVILYDNMGAGTTNPEYFDFDRYSSLEGYAYDLISILDELQVHSCIFVGHSVSGMVGVIASVTRPDLFSKLVMIAASPRYLNDVDYFGGFEQEDLDQLFDAMGKNYKAWCSGFAPLAVGGDMDSVAVQEFSRXGQTIFQSDMRQILGLVSVPCHILQSFKDLAVPIVISEYLHQHLGGESIVEVMSSEHLPQLSS

**MpKAI2**

CCTGAAGTACAAACATTATTGGCATCTAATCCTCTTGTTATTTTTTATCTGCATATTTATMGIAAEAHNVKILGSGTEYIVLAHGFGTDQSLWKHFVPYLIDDYRVILYDNMGAGTTNPDYFDFERHSSLEGYASDLLAILEELHVESCIFVGHSVSAMIGAIASISRPDLFAKLIMVGASPRYLNDVEYYGGFEQEDLNQLFDAIAANYKAWCYGFAPLAVGGDMDSVAVQEFSRTLFNMRPDIALIVSRTIFQSDMRQILSLVTVPCHIIQAEKDMAVPVMISEYLHQHLGAESIVEVMTTDGHLPQLSS

**MtKAI2**

CCAAAACATATCCGAATAAAAACACAAAATGAAAGAAAAAAAAAAAGAAGGGAAAAAACAMGIVEEAHNVKVLGSGSRFIVLAHGFGTDQSVWKHLVPHLLDEFRVILYDNMGAGTTNPDYFDFERYSTLEGYAYDLLAILEELRVDSCIFVGHSVSAMIGTVASISRPDLFNKIILISASPRYLNDRDYFGGFEQEDLDQLFDAMASNYKSWCSGFAPMAVGGDMESVAVQEFSRTLFNMRPDIALSVLQTIFKSDMRQILCMVTVPCHIIQSMKDLAVPVVVAEYLHQHVGSESIVEVMSTEGHLPQLSS

**NaKAI2**

CTAAGACATGGAAGAAAGGAAGAGAATACGTTGTCACGTGATTAGCACATGGACATTCTTMGIVEEAHNVKILGSGEQTVVLAHGFGTDQSVWKHLVPHLVDDYKIILFDNMGAGTTNPDYFDFERYSSLEGYAYDVIAILEDLKIPGCIYVGHSVSAMIGVIASIARPDLFTKLVTVSASPRYLNDSDYYGGFEQEDLDQLFEAMRSNYKAWCSGFAPLVVGGDMDSVAIQEFSRTLFNMRPDIALSVLQIIFLSDLRHLLAHVTVPCHLIQSMKDLAVPVVVSEYLHQHLGGKSIVEVISTEGHLPQLSS

**PaKAI2**

ATCCTAACCCAAAATATCATATCATTACATTATAATCTAAAAAGTCTAAACCAACAATGTMGIVEEAHNVRVLGSGQQVIVLAHGFGTDQSVWKHLVPHLIDDYRVIMYDNMGAGTTNPEYFDFERYATLEGYAYDLLAILEELRVGSCIFVGHSVSGMVGAIAAITRPDLFTKLVMIGASPRYLNDVDYYGGFEQEDLEQLFDAIRSNYKAWCSGFAPMAVGGDLDSVAVQEFSRTLFNMRPDIALSVAQTIFQSDTRQILHLITVPCHILQSVKDLAVPVVVTEHLHQNLGGESIVEVMSSDGHLPQLSS

**PeKAI2**

CCACAAATTATTCTCCAAGTATCAACATCTCCATTCTTGTAACCCCATTTACTAGTTGTTMGIVEEAHNVKILGSGERVIVLAHGFGTDQSVWKHLIPHIVDEYKVILYDNMGAGTTNPDYFDFNRYSSLEGYAYDLLAILEELKVESCILVAHSVSCIIGAIASISRPDLFSKIVMLSASPRYLNDVDYYGGFEQEDLDQLFEAMQNNYKAWCSGFAPLAVGGDMDSVAVQEFSRTLFNMRPDIALSVAQTIFQSDMRSILHMVTVPCHILQSMKDLAVPVVAAEYLHQNLGGESIVEVMSSDGHLPQLSS

**PpKAI2**

ACTGTAGCAACTCTCCTAACTTACCCCAATTGACTCCCAAGTTTTGCACAACACAACACTMGIVEEAHNLRVLGSGQQVIVLAHGFGTDQSVWKHLVPHLVDDYRVVMYDNMGAGTTNPEYFDFERYATLEGYAYDLLAILEELRIGSCIFVGHSVSGMVGAIAAITRPDLFTKLVMVGASPRYLNDVDYYGGFEQEDLEQLFDAIRSNYKAWCSGFAPMAVGGDLDSVAVQEFSRTLFNMRPDIALSVAQTIFQSDTRQILHLITVPCHILQSVKDLAVPVVVTEYLHQNLGGESIVEVMSSDGHLPQLSS

**PyKAI2**

CACCGCGAAAGGGCTCGCCCGAGGACCTGAGCTAGCATGGTTGGAGCACTTAAATGCGGCMGIVEEAHNVRVLGSGQQVIVLAHGFGTDQSVWKHLVPHLVDDYRVVMYDNMGAGTTNPEYFDFERYATLEGYAYDLLAILEELRVGSCIFVGHSVSGMVGAIAAITRPTSSPSSYLNDVDYYGGFEQEDLEQLFDAIRSNYKAWCSGFAPMAVGGDLDSVAVQEFSRTLFNMRPDIALSVAQTIFQSDTRQILHLITVPCHILQSVKDLAVPVVVTEYLHQNLGGESIVEVMSSDGHLPQLSS

**RcKAI2**

AAATGGCCCTCCGTACTTAATAACTCCACCGTGTTCCGGCCACCCAAATGGGTCTTCAAAMGIVAEAHNVRVLGSGQQVIVLAHGFGTDQSVWKHLVPHLVDDYRIVMYDNMGAGTTNPEYFDFERYSTLEGYAYDLLAILEEFQVESCIFVGHSVSAMIGTIAAVMRPELFFKLVMIAASPRYLNDTDYYGGFDEEDLQQLFAIRSNYKAWCAGFAPLAVGGDMDSVAVQEFSRTLFNMRPDIALSVAQTIFQSDTRHIMGMVTVPCHILQSVKDLAVPVVVSEYLHQNLGGVSIVEVMSSDGHLPQLSS

**SlKAI2**

TACTCGGCCACCGATGCTGGGACAGACACGTCTTTCGCTGTCTGAATTATACAACAAGGTMGIVEEAHNVKILGTGDRSIVLAHGFGTDQSVWKHLVPHLVEDYKVVLFDNMGAGTTNPDYFDFERYSTLEGYAYDVIAILEELQIPCCIYVGHSVSAMIGAIASVARPDLFTKLVTVSGSPRYLNDVDYYGGFEQEDLDQLFEAMRSNYKAWCSGFAPLAIGGDMDSVAVQEFSRTLFNMRPDMALSVLQIIFQSDLRHMLPHVTVPCHIIQSMKDLAVPVVVSEYLHQNLGGESIVEVMSTDGHLPQLSS

**SpKAI2**

ACCTTAACTAGGCCCAATATTCCTCTTAGATCACTATTAAAAACTGTTCTCGACACAAACMGIVEEAHNVKILGSGEQTVVLAHGFGTDQSVWKHLVPHLIDEYRVVLFDNMGAGTTNPDYFDFERYSSLEGYAYDVIAILEEFQIRSCIFVGHSVSAMVGAIASIARPDLFTKIVTVSASPRYLNDSEYYGGFEREELDQLFEAVKTNYKSWCSGFAPLVVGGDMDSVAIQEFSRTLFNMRPDIALSVIQIIFLSDLRHLLPHVSVPFHIIQSMKDLAVPVVVSEYLSQNLGSESIVEVMSTEGHLPQLSS

**VuKAI2**

ACATCCGCCCCCACCGCCAGCGGCGCAAAACCGTTCACCCACGCCTCATAATTCGCCTCCMGIAAEAHNVKILGSGTEYIVLGHGFGTDQSVWKHFVPYLVDDYRVVLYDNMGAGTTNPEYFDFERYATLEGYASDLLAILEELQVESCIFVGHSVSGMVGAIASIARPDLFTKLVMVGASPRYLNDVDYYGGFEQEELNQLFDAMATNYKAWCCGFAPLAVGGDLESVAVQEFSRTLFNMRPDIGLMVSRTIFQSDMRHILNLVSVPCHIIQAEKDMAVPVVISEYLHQNIAAESIVEVVPTEGHLPQLSS

**VvKAI2**

ATTGCAGTAAAAACATTCTCTATATCCGCTCGATCGAATCCGCCGTGGTAATCTTTGTCAMGIVEEAHNLKVVGSGEQIIVLAHGFGTDQSLWKHLVPHLVDDYRVILFDNMGAGTTNPEYFDFERYSNLEGYAYDVLAILEELQVQSCIFVGHSVSAMIGAIASITRPDLFSKLISINGSPRYLNDVDYYGGFEQEDLDQLFEAMGSNYKAWCSGFAPLAVGGDMDSVAVQEFSRTLFNMRPDIALSVAQTIFQVDLRQILCHVTVPCHILQSIKDLAVPVVVSEYLHQNLGGESIVEVMTSDGHLPQLSS

**ZjKAI2**

TTCAAGAACCTGGGAGAAGCGCCGATGAGGATGAGTTTGGAGAAAAGTTCAGGGCGTCTAMGILEEAHNLKVVGTGQQILVLGHGFGTDQSVWKHLIPHIIDDYKVILYDNMGAGTTNPDYFDFERYSTLEGYAYDLLAILEELQVDSCIYVGHSVSAMVGLIASITRPDLFSKIIMIAASPRYLNDVDYYGGFEQEDLDQLFEAMGANYKAWCSGFAPLAVGGDMDSMAVQEFSRTLFNMRPDIALTVAQTIFQSDMRQILKMVTVPCHILQSVKDLAVPVVVSEYLHQNLGGESIVEVMSSDGHLPQLSS

**List of genes used in manuscript**

***SsKAI2* full sequence with translated ORF**

cgatctcttatcatcccccgtcgacaccctttgcgtccacaataacattagcagccaaaagaaagtccactttttca

tactattaaatcccctaattcccccgcaatccaagaaactaacttggaccaaaatcgagaaatccataaacaagaa

atgggtatagtagaagaagctcataacctcaagattttgggcactggtgctcaggtcttg

M G I V E E A H N L K I L G T G A Q V L

gtcctcgctcatgggtttggtactgatcagtctgtgtggaaacaccttgtccctcatctt

V L A H G F G T D Q S V W K H L V P H L

gttgatgattttaaggttgtcttgtttgataatatgggtgctggtactactaacccagat

V D D F K V V L F D N M G A G T T N P D

tactttgatttcgagagatatgccagtcttgaaggatttgcttatgatttgcttgccatt

Y F D F E R Y A S L E G F A Y D L L A I

ttggatgaattgcaaattgaatcatgtatttttgtgggtcattctgtttctgctatggtt

L D E L Q I E S C I F V G H S V S A M V

ggtgctattgcctccatatctcgcccagatcttttctccaaaatcatcatgctctctggt

G A I A S I S R P D L F S K I I M L S G

tccccaaggtatttgaacgatgtggattactatggaggatttgagaaagaagatctaaac

S P R Y L N D V D Y Y G G F E K E D L N

caactatttgatgcaatgcaatcgaattacaaggcttggtgcgcagggtttgcgccgttg

Q L F D A M Q S N Y K A W C A G F A P L

atggtgggtggagatatggattcagtggtggttcaagaattcagccgcactctctttaat

M V G G D M D S V V V Q E F S R T L F N

atgagaccagacatagccttatctgtggcacaaactatatttcaaacagatatgcgacaa

M R P D I A L S V A Q T I F Q T D M R Q

attctccatctggtgatagtgccttgtcatattctgcaaagttctacggacttagctgtg

I L H L V I V P C H I L Q S S T D L A V

ccagtagttgtgtctgaatatttgcataagcaccttggtggtgagtctattgtagaaata

P V V V S E Y L H K H L G G E S I V E I

ataccgacggatggtcacttgccgcaattgagctcgccagatatttggattccggctctt

I P T D G H L P Q L S S P D I W I P A L

Cttagacacatacgacacgatattgccacttgatttagtattggattttatatttactatggtcaatttg

L R H I R H D I A T -

Tgtgtgctttttagtattggattttatatttactatggtcaatttgtgtgtgcttattaggttattaattccttaatggaac

Tacataaattgttaattaggattatggaagctatcaagctaatggagtaaaattgtgtgactataaaatcatttgtgt

Ctgcctttaatgtcaactatatagatgaataagagtattctgcaatatgcatgaagaattaaaa

***AtCSP1*** [***AT4G36020***](https://www.araport.org/locus/AT4G36020)

***AtCSP2*** [***AT4G38680***](https://www.araport.org/locus/AT4G38680)

***AtCSP3*** [***AT2G17870***](https://www.araport.org/locus/AT2G17870)

***AtCSP4*** [***AT2G21060***](https://www.araport.org/locus/AT2G21060)

***AtCBF1*** [***AT4G25490***](https://www.araport.org/locus/AT4G25490)

***AtCBF2*** [***AT4G25470***](https://www.araport.org/locus/AT4G25470)

***AtCBF3*** [***AT4G25480***](https://www.araport.org/locus/AT4G25480)

***AtCBF4*** [***AT5G51990***](https://www.araport.org/locus/AT5G51990)

***AtNCED3*** [***AT3G14440***](https://www.araport.org/locus/AT3G14440)

***AtAAO3*** [***AT2G27150***](https://www.araport.org/locus/AT2G27150)

***AtABA1 AT5G67030***

***AtCYP707A1*** [***AT4G19230***](https://www.araport.org/locus/AT4G19230)

***AtCYP707A2*** [***AT2G29090***](https://www.araport.org/locus/AT2G29090)

***AtCYP707A3*** [***AT5G45340***](https://www.araport.org/locus/AT5G45340)

***AtABI3***  [**AT3G24650**](https://www.arabidopsis.org/servlets/TairObject?id=38924&type=locus)

***AtABI5*** [**AT2G36270**](https://www.arabidopsis.org/servlets/TairObject?id=32860&type=locus)

***AtABF1*** [**AT1G49720**](https://www.arabidopsis.org/servlets/TairObject?id=27527&type=locus)

***AtMYB3R2*** [**AT4G00540**](https://www.arabidopsis.org/servlets/TairObject?id=128318&type=locus)

***AtMYB96*** [**AT5G62470**](https://www.arabidopsis.org/servlets/TairObject?id=131919&type=locus)

***AtSIZ1***  [**AT3G25910**](https://www.arabidopsis.org/servlets/TairObject?id=38740&type=locus)

***AtSnRK2.3*** [**AT5G66880**](https://www.arabidopsis.org/servlets/TairObject?id=134777&type=locus)

***AtACTIN2*** [***AT3G18780.2***](https://www.arabidopsis.org/servlets/TairObject?type=gene&id=1000639330)
